# Evolution and New Perspectives of Balloon Pulmonary Angioplasty in CTEPH

**DOI:** 10.3390/jcm14030699

**Published:** 2025-01-22

**Authors:** Julia Larsen, Vladimir Lakhter, Amine Nasri, Riyaz Bashir

**Affiliations:** Department of Cardiovascular Diseases, Temple University Hospital, Philadelphia, PA 19140, USA; julia.larsen@tuhs.temple.edu (J.L.); vladimir.lakhter@tuhs.temple.edu (V.L.);

**Keywords:** chronic thromboembolic pulmonary hypertension (CTEPH), chronic thromboembolic pulmonary disease (CTEPD), balloon pulmonary angioplasty (BPA)

## Abstract

Chronic thromboembolic pulmonary disease (CTEPD) and chronic thromboembolic pulmonary hypertension (CTEPH) are debilitating complications of acute pulmonary embolism (PE) that are characterized by fibrosis and organization of the thrombotic material within pulmonary artery branches. This pathology leads to increased right ventricular afterload and dead space ventilation, posing a risk of progressive pulmonary hypertension, right-sided heart failure, and potentially death if left untreated. Pulmonary endarterectomy (PTE) is a technically complex open-heart surgery considered to be a first-line treatment as it is a potentially curative therapy. Although PTE is highly successful in proximal disease, it may not reach the very distal branches. On the other hand, pulmonary vasodilator therapy is very effective in improving microvasculopathy but does not address the obstructive fibrotic component of the larger vessels. Balloon pulmonary angioplasty (BPA) is a novel percutaneous revascularization therapy in which traditional angioplasty techniques are used to relieve obstruction in the pulmonary arteries. This review discusses the currently accepted indications, patient selection, technical considerations, outcomes, and complications of contemporary BPA. This review will address knowledge gaps and future perspectives in BPA research.

## 1. Introduction

Chronic thromboembolic pulmonary disease (CTEPD) and chronic thromboembolic pulmonary hypertension (CTEPH) are underdiagnosed and undertreated complications of acute pulmonary embolism (PE). They are caused by the fibrotic organization of the thrombotic material in pulmonary vessels, leading to increased afterload of the right ventricle (RV) and an increase in dead space ventilation. When this pathology causes an increase in resting mean pulmonary arterial pressure (mPAP) of greater than 20 mmHg or a pulmonary vascular resistance (PVR) > 2 Wood Units (WU) with normal capillary wedge pressure (≤15 mmHg), the condition is called CTEPH; whereas, it is called CTEPD if the resting mPAP is less than 20 mmHg [1]. These patients can present with a variety of symptoms, which include progressive dyspnea, exercise intolerance, fatigue, chest discomfort, dizziness, palpitations, and syncope [2,3]. If left untreated, patients with CTEPH are at risk of progressive pulmonary hypertension, right-sided heart failure, and potentially death [1,4,5]. The overall incidence of CTEPH after an acute PE has been reported to range from 2 to 9%, and the prevalence ranges from 26 to 38 cases/million adults [1]. On the other hand, CTEPD incidence is estimated to be much higher, approximately 16% among survivors within two years of the index intermediate- and high-risk PE event [1].

Current treatment modalities for the management of CTEPD/CTEPH include pulmonary endarterectomy (PTE), balloon pulmonary angioplasty (BPA), and pulmonary hypertension (PH)-targeted medical therapy. The first-line and potentially curative therapy for these patients is PTE. Nevertheless, PTE is a highly invasive and morbid surgery involving median sternotomy, deep hypothermia, and circulatory arrest. This operation removes organized and fibrotic thrombi from surgically accessible pulmonary vessels. However, one-third of these patients have isolated distal disease, which is not surgically accessible, and up to 40% of patients with technically operable lesions are excluded from PTE due to high surgical risk, patient refusal, or a lack of access to advanced surgical facilities [2]. Patients with residual or recurrent pulmonary hypertension after prior PTE surgery are poor candidates for repeated PTE.

Medical therapy for PH aims to cause pulmonary vasodilation and reduce vascular cell proliferation by targeting one of three key pathways: nitric oxide (NO), endothelin, and prostacyclin [6]. Riociguat, a soluble guanylate cyclase (sGC) stimulator acting on the NO pathway, remains the only approved medical therapy for patients with inoperable CTEPH or persistent pulmonary hypertension post-PTE surgery [3,7]. Riociguat effectively improves microvasculopathy but fails to address the large vessel obstructive fibrotic component of CTEPD [2]. Furthermore, Riociguat therapy for pulmonary hypertension necessitates a lifetime of expensive treatment with a frequently intolerable side effect profile [2,8,9]. For patients with small peripheral vessel occlusions that are inaccessible for PTE or BPA, medical therapy remains the best therapeutic option [10]. Because both Riociguat, a sGC stimulator, and phosphodiesterase type 5 inhibitors such as Sildenafil and Tadalafil act on the NO pathway, coadministration is contraindicated due to the increased risk of systemic hypotension [6]. Other medical therapies include the endothelin inhibitor Bosentan and prostanoids such as Epoprostenol and Iloporost [3,11,12,13]. It has been shown that 5-year survival rates for patients undergoing both PTE and BPA are around 98% [14]. By comparison, historical survival rates for CTEPH patients treated with medical therapy alone are closer to 64% [15,16].

Balloon pulmonary angioplasty is a novel percutaneous revascularization therapy in which traditional angioplasty techniques relieve obstruction in the pulmonary arteries, thereby improving pulmonary vascular blood flow, reducing RV afterload, and improving dead space ventilation [2,17].

History of Balloon Pulmonary Angioplasty: In 1988, Voorburg and colleagues published the first case report of BPA in the treatment of inoperable CTEPH [18,19]. In 2001, Feinstein and colleagues expanded upon this initial finding with a larger cohort study (n = 18) of BPA therapy and found significant improvement in several hemodynamic and functional parameters [18,20]. However, there was a high rate of major complications, namely, reperfusion pulmonary edema (61.1%), endotracheal intubation (16.6%), and death (5.5%), resulting in poor adoption of this therapy within the United States [18,20]. The recent acceptance of BPA as a feasible treatment option is largely due to the advancements made by several Japanese groups who have refined procedural techniques such as using undersized balloons in initial sessions and intravascular ultrasound to ascertain appropriate balloon size and pressure wire guidance [21,22,23]. Such refinements have led to significant improvements in the safety and clinical outcomes of BPA. The 2022 European Society of Cardiology (ESC)/European Respiratory Society (ERS) guidelines for the diagnosis and treatment of pulmonary hypertension have recommended BPA as class I treatment for patients with inoperable CTEPH [1].

However, this recommendation is based mainly on observational data and two randomized controlled trials (RCTs) from Japan and Europe. Due to significant CTEPH treatment heterogeneity worldwide, a large multicenter RCT in the U.S. is necessary before wide-scale adoption in this country. This review discusses the currently accepted indications, patient selection, technical considerations, outcomes, and complications of contemporary BPA. It will also address knowledge gaps and future perspectives in BPA research.

## 2. Patient Evaluation and Pre-BPA Planning

Patients with CTEPH often present with progressive dyspnea and exercise intolerance and may exhibit symptoms of right ventricular hypertrophy or failure as the disease progresses [3]. As such, initial diagnostic tests include chest radiography, pulmonary function studies, and electrocardiography to rule out more common cardiopulmonary causes [3]. Ventilation/perfusion (V/Q) lung scan is the best first test used to identify mismatched perfusion defects. The sensitivity of this test is very high (97.4%) in detecting perfusion abnormalities, and the absence of perfusion defects essentially rules out CTEPD as a suspected cause of dyspnea (negative predictive value of nearly 100%) [3,24]. However, the low specificity (90%) and positive predictive value (83.5%) of an abnormal V/Q finding necessitate further imaging to confirm the diagnosis of CTEPD [24]. The location of these perfusion defects on V/Q scans can be a helpful guide to the revascularization of various BPA targets [17]. CT pulmonary angiography is critical in the confirmation of CTEPD/CTEPH diagnosis. It also identifies lesion characteristics and an underlying parenchymal lung or mediastinal disease and rules out other pulmonary vascular disorders that could present as perfusion defects on a V/Q scan [3]. Cardiac magnetic resonance (CMR) offers a comprehensive assessment of pulmonary vascular anatomy while simultaneously evaluating cardiopulmonary hemodynamics and RV function [3,25].

## 3. Catheter-Based Pulmonary Angiogram

**Figure 1:**
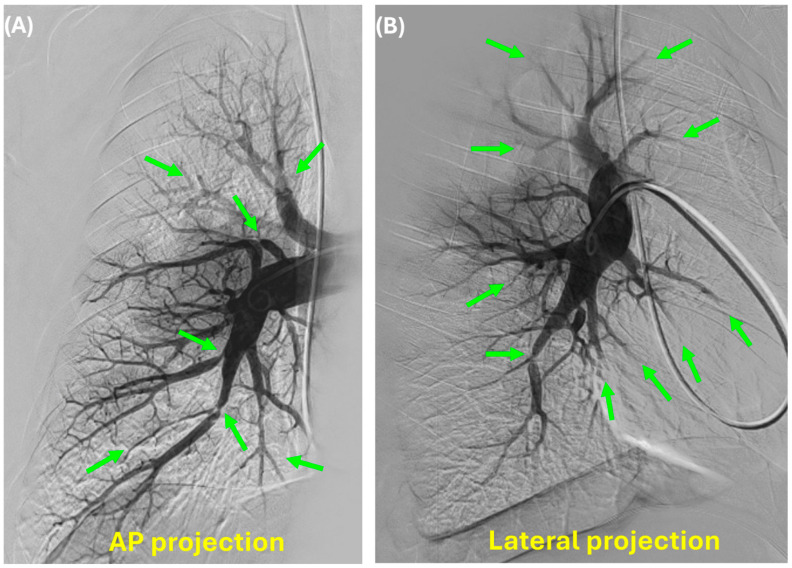
Example of pulmonary angiogram of the right pulmonary artery; AP projection (**A**) and Lateral projection (**B**) are shown. Green arrows reveal areas of pulmonary vascular obstruction.

**Figure 2:**
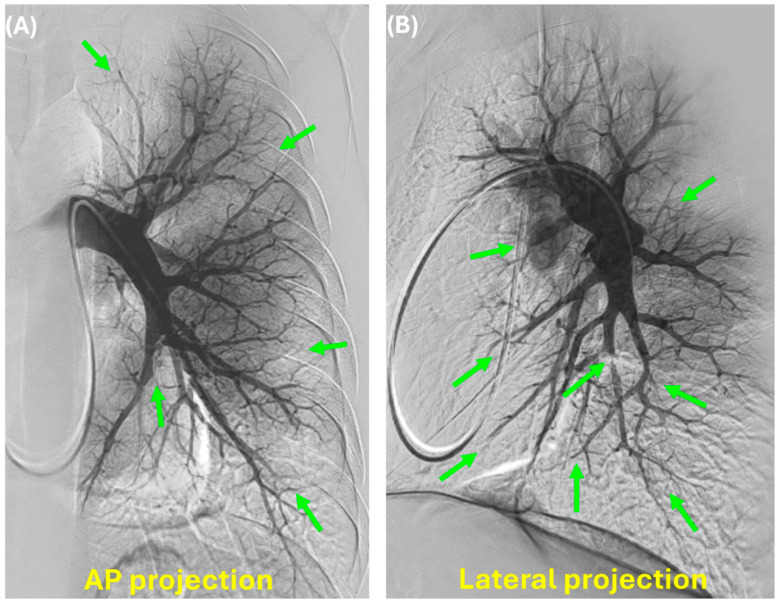
An example of a pulmonary angiogram of the left pulmonary artery; the AP projection (**A**) and lateral projection (**B**) are shown. Green arrows reveal areas of pulmonary vascular obstruction.

Catheter-based pulmonary angiography (CPA) alongside right-heart catheterization has been considered the “gold standard” for evaluating CTEPD as it can confirm the presence of perfusion defects and localize and characterize lesions suitable for BPA therapy [3]. CPA for CTEPD assessment is performed by introducing a 7 Fr vascular sheath into one of the internal jugular or femoral veins. This is followed by a right heart hemodynamic assessment using a standard balloon-tipped, flow-directed catheter (Swan Ganz). Then invasive PA angiography of each pulmonary artery is performed using digital subtraction with breath hold in deep inspiration [26]. The Berman catheter or the 7F high-flow pigtail catheter is placed in the right and the left main pulmonary artery. Imaging is usually performed in an AP or 15° ipsilateral oblique and lateral views on each side, ideally with a biplane detector to reduce the contrast load (Figure 1 and Figure 2). The catheter tip is optimally positioned within the descending pulmonary artery, distal to the lower-lobe superior segment, so that the middle and lower-lobe vessels can be visualized concurrently with rapid contrast backfilling into upper-lobe vessels. The contrast agent most often used is non-ionic, isosmolar iodixanol (Vispaque; GE Heathcare, Marlborough, MA, USA) [26,27]. The author uses the same pigtail to perform pelvic venous and inferior vena cava angiography to assess any pelvic venous pathology that may have led to deep vein thrombosis (DVT) in the first place or may have caused post-thrombotic syndrome of the lower extremities. A pelvic venous angiogram is also helpful in choosing a proper access site if the patient needs BPA.

## 4. Indications and Patient Selection for BPA

After confirming the CTEPH/CTEPD diagnosis, operability should be rigorously assessed by a comprehensive, multidisciplinary team consisting of PTE-experienced surgeons, radiologists experienced in pulmonary vascular imaging, pulmonary vascular medicine specialists, and interventional cardiologists experienced in BPA [3,4,28]. Given that operability is heavily influenced by local surgical expertise, patient comorbidities, location of lesions, and informed patient preference, it is recommended that eligibility assessment for BPA and the procedure only be performed in experienced, high-volume CTEPH centers [3,17]. As specified by a clinical consensus statement of the European Society of Cardiology Working Group on Pulmonary Circulation, BPA centers should perform a minimum of 100 procedures/year and be able to demonstrate excellent safety outcomes with a 30-day mortality of <2% and serious adverse event rates of <5% per session [29].

**Figure 3:**
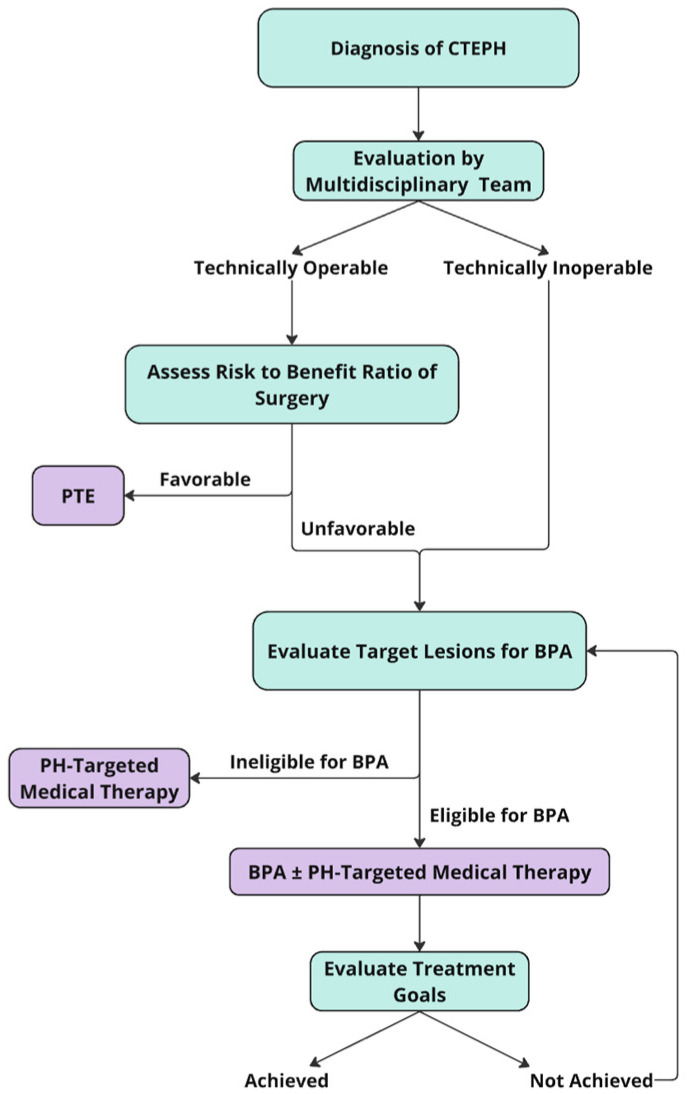
A flow diagram illustrating a treatment algorithm for patients diagnosed with CTEPH. Abbreviations: CTEPH: chronic thromboembolic pulmonary hypertension; PTE: pulmonary thromboendarterectomy; PH: pulmonary hypertension; BPA: balloon pulmonary angioplasty.

PTE remains the first-line therapy and is a potential cure for CTEPH patients; therefore, an experienced multispecialty team, which includes an experienced PTE surgeon, must first assess the patient for operability. Only patients who have inoperable disease or operable disease with excessive surgical risk should be considered for BPA, as well as patients with residual or recurrent pulmonary hypertension despite prior PTE operation (Figure 3). Patients who refuse PTE after fully understanding the risks and benefits of both PTE and BPA can be also considered for BPA [13,30]. Patients with iodine or contrast allergy need to be pretreated before the procedure [29]. It is important to note that the selection of patients for BPA is not well standardized, and a patient’s operability status is highly dependent on local surgical expertise as it relates to patient comorbidities, disease location, severity of vascular occlusion, lesion types, hemodynamic impairment, and patient preferences [3,17,31]. Significant predictors of improvement in 6MWD with BPA include young age, tall stature, high mean pulmonary artery pressure, short 6MWD at baseline, and high baseline lung capacity [32].

Special categories of patients must be considered when planning BPA procedures due to various comorbidities and anatomical differences. Although a typical treatment regimen involving multiple BPA procedures exposes a patient to a considerable amount of contrast, studies have shown that BPA can be safely performed in patients with chronic kidney disease [29,33,34]. BPA is also a feasible therapeutic option in patients experiencing residual PH after PTE, which occurs in 17–31% of patients [29,35,36,37]. BPA post-PTE is efficacious in improving hemodynamics and functional class but may have slightly higher rates of adverse events, so rigorous multidisciplinary assessment of these patients 3–6 months after PTE is crucial [29]. Some patients may have one lung with operable CTEPH, and the contralateral lung has an inoperable, distal disease. Such patients can be considered for a stepwise approach with PTE and follow-up treatment with BPA. However, these patients have a significant perioperative risk for complications and death, particularly in patients with severely abnormal resting hemodynamics [38]. In these cases, patients at centers experienced in both therapeutic techniques may be considered for hybrid procedures in which PTE and BPA are concurrently performed, which has been shown to reduce PVR [38]. In patients with Takayasu Arteritis, percutaneous pulmonary angioplasty strategies, including stent placement in proximal lesions, have been associated with reduced risk of all-cause mortality and acceptable periprocedural risk in patients with pulmonary artery hypertension [39]. Individual variations of pulmonary arterial anatomy also need to be considered when planning BPA to prevent vascular injury [29,40]. In challenging cases, 3D printing and augmented reality may assist in guiding transcatheter pulmonary interventions by elucidating spatial relationships more accurately than conventional angiography [37,41].

## 5. Technical Considerations

**Figure 4:**
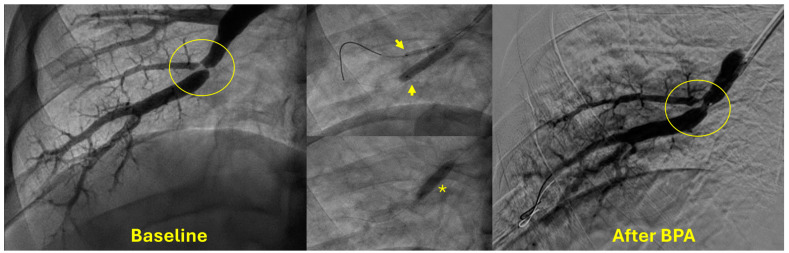
An example of balloon pulmonary angioplasty of a severe lesion (yellow circle) at a bifurcation of the anterior (A8) right lower lobe segmental artery. Kissing balloon angioplasty (yellow arrows) followed by angioplasty of the common vessel (asterix) was used for treatment.

The most common access site is the femoral vein (right or left). However, internal jugular veins can be used in patients with inferior vena cava or bilateral iliac vein occlusions. Compared to the jugular approach, the femoral approach is associated with increased operator comfort and reduced radiation exposure. After placing a venous sheath using an ultrasound-guided micropuncture system (usually a 9F short sheath), right heart catheterization is performed using a 7F Swan Ganz catheter with a 0.035″ guidewire lumen. The catheter is then exchanged over a 0.035″ Storq wire for a 7F 70-cm long sheath whose distal tip is positioned in the lobar artery. Through this long sheath, a 6F guiding catheter is used to cannulate segmental vessels selectively and identify lesions by performing selective angiography. Then, a 0.014″ coronary wire is used to cross the lesion. Guidewires with soft, atraumatic tips are usually selected first, while polymer-jacketed wires are usually avoided to reduce the risk of perforation. Balloon pulmonary angioplasty is then performed over the 0.014″ wire with undersized noncompliant balloons. At times, simultaneous “kissing-balloon angioplasty” is required to treat lesions at a site of a bifurcation (Figure 4).

**Figure 5:**
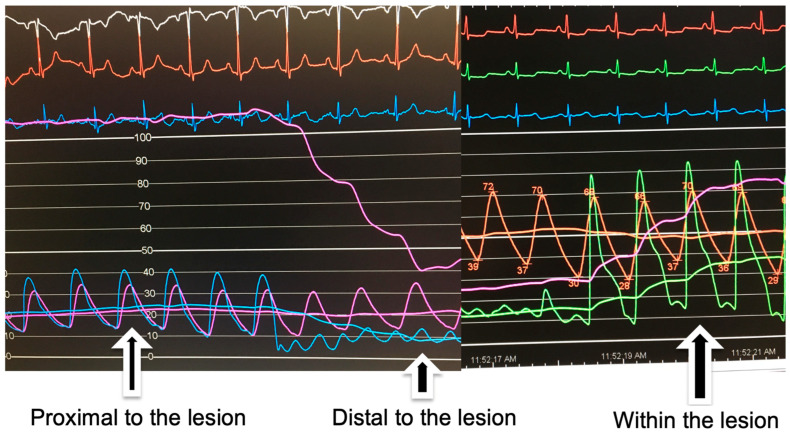
Hemodynamic guidance of balloon pulmonary angioplasty using Radi wire. Proximal to the lesion, both the guide pressure (purple tracing) and the Radi wire pressure (blue tracing) are the same. After the wire crosses distal to the lesion, the Radi wire pressure (blue and green tracings) drops below the guide pressure (purple and orange tracings) and has a wedge pressure-like appearance. When the Radi wire pressure tip is positioned right at the lesion, the Radi wire pressure (green tracing) has a “ventricularized” waveform.

Various guiding catheters can be used for selective cannulation of segmental pulmonary arteries; the JR4 or MP guiding catheter is usually preferred for the right lung, while the AL1 guiding catheter is preferred for the left lung. Another variation in the BPA technique is using a pressure wire to guide balloon pulmonary angioplasty. A pressure wire can help localize a lesion when it is not easily seen on angiography (Figure 5). It also reduces the risk of reperfusion pulmonary edema (RPE) by keeping the pressure distal to the lesion less than 35 mmHg mean [23].

At times, accessing the right lung and right pulmonary artery may be challenging secondary to vessel tortuosity; in such cases, using a pigtail catheter and a hydrophilic-coated guidewire (Glidewire) can be helpful. The sheath can then be advanced over the pigtail (used as a dilator) into the right PA. For very proximal lesions, intravascular ultrasound (IVUS) guidance is preferred for proper balloon sizing of the vessels. However, an 8F sheath is required to advance a 0.035″ IVUS catheter; this cannot be performed through a 6F guiding catheter. Selective angiography is then performed to localize the pulmonary vascular lesions; in many cases, a deep breath hold by the patient may be helpful to cannulate the vessel or cross the lesion with a wire. In certain cases, where angiography does not clearly demonstrate a lesion, a pressure wire can help localize a stenosis and determine its hemodynamic significance. Some operators also use a guide extension catheter to better visualize the distal branches and to augment balloon delivery. Ostial lesions are a challenge and may require two wires: one to maintain the guide catheter position and another one for crossing the lesion. The use of a pressure wire for these ostial lesions helps confirm the presence of a pressure gradient and differentiate stenosis from vessel tortuosity.

In the presence of bilateral disease (majority of treated patients), the authors prefer to treat only one lung during a single session. We frequently treat the lower lobes first and work our way up to the middle/lingular and upper lobes. The authors recommend intentionally undersizing the balloons relative to the vessel size (usually starting with a 2.0 mm or a 2.5 mm balloon) during the initial treatment session in order to reduce the risk of procedure-related complications (i.e., pulmonary vascular injury and reperfusion pulmonary edema). During subsequent sessions, we frequently re-treat these same areas with larger balloons (sized closer to 1:1) to optimize vessel perfusion. In order to reduce the risk of contrast-induced nephropathy, we use diluted contrast to perform selective pulmonary angiography. For patients with significant chronic kidney disease, calculation of a maximum allowable contrast dose (MACD: 5 mL × body weight in kg/baseline serum creatinine in mg/dL) can also be useful in reducing the risk of renal injury.

**Figure 6:**
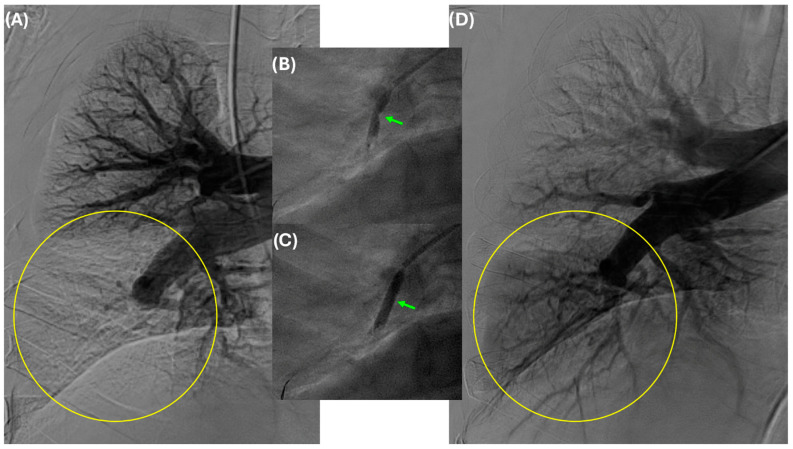
Balloon pulmonary angioplasty of a right distal interlobar occlusion (panel **A**, yellow circle). Upon the initial phase of balloon inflation, there is a “dog bone” appearance of the balloon indicating the location of the stenosis (panel **B**, green arrow). As the balloon continues to inflate, it can modify the stenosis and fully expand (panel **C**, green arrow). After angioplasty, there is restoration of flow and markedly improved perfusion of the lower lobe pulmonary artery (panel **D**, yellow circle).

**Figure 7:**
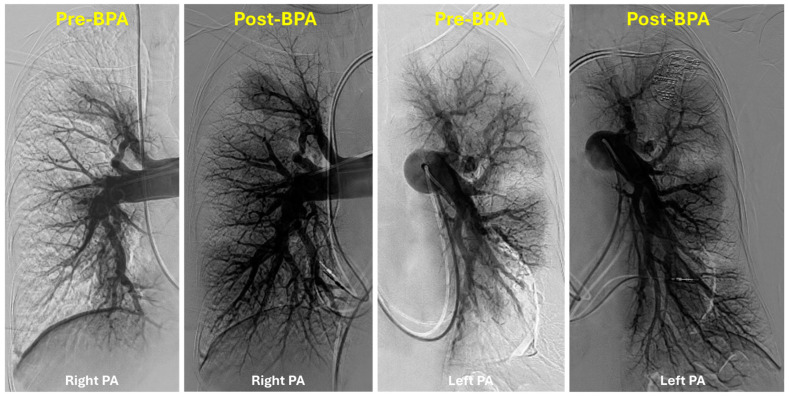
Non-selective pulmonary angiography of the right and left pulmonary arteries pre- and post-balloon pulmonary angioplasty: there is a marked improvement in perfusion in all zones after completion of balloon pulmonary angioplasty.

The authors prefer to treat chronic total occlusions after the mean pulmonary artery pressure is initially decreased following several BPA sessions (Figure 6). A determination whether to stop further BPA treatments is usually made either hemodynamically, angiographically (no further treatable lesions remain), or using a combination of both (Figure 7). Key predictors of the total number of BPA sessions include the severity of pulmonary hypertension (high mPAP), baseline exercise capacity (low 6MWD), patient age, patient sex, the extent of vascular obstruction in the lungs, and the presence of significant right ventricular dysfunction [42].

## 6. Outcomes

A recent meta-analysis (n = 1763) of BPA outcomes showed an improvement in most of the functional and hemodynamic parameters following BPA. Specifically, mPAP decreased by 13.2 mmHg (95% CI −14.7 to −11.8; *p* < 0.001), PVR decreased by 3.89 WU (95% CI −4.38 to −3.39; *p* < 0.001), 6MWD improved by 70 m (95% CI 58 m to 82 m; *p* < 0.001), and WHO functional class improved by an average of one class (95% CI −1.2 to −0.9; *p* < 0.001) [43]. Similar results were seen in patients who underwent BPA post-PTE [43]. A multicenter registry of patients with inoperable CTEPH who underwent BPA at 7 Japanese centers (n = 308; 1408 procedures; 80% female) reported a 3-year survival of 94.5% and a 44% reduction in mPAP (43.2 ± 11.0 mm Hg to 24.3 ± 6.4 mm Hg after final BPA) [3,44]. Although limited, contemporary U.S. data (Table 1) show similar improvements in these outcomes. It is crucial to note that these results might not be universally applicable and could be replicable only in specialized centers, given the inherent complexities of CTEPH management [18]. Even in expert BPA centers, there is a significant learning curve associated with the BPA procedure; as reported by Brenot et al., periprocedural complications decreased from 13.3% to 5.9% from their early cohort to the later cohort [18,45].

## 7. Global Heterogeneity of Patient Selection for BPA

It is essential to recognize that most evidence for BPA comes from institutions outside of the U.S. and patient populations may not reflect U.S. demographics. Therefore, caution should be exercised when applying BPA findings domestically. A worldwide prospective CTEPH Registry of 1010 patients found that 75% of Japanese CTEPH patients were female, whereas gender was evenly balanced in Europe and the United States [50]. The median age was similarly heterogeneous as Japanese and European patients were ~10 years older at diagnosis than U.S. patients [50]. Furthermore, 70% of Japanese patients are treated with BPA. In comparison, PTE is the treatment of choice for 75% of U.S. patients, which may reflect differing experience levels with various therapeutic modalities in these countries [2,50,51]. Such stark regional differences in demographics and operability of CTEPH need to be considered while interpreting the observational BPA data [2].

## 8. Findings from Randomized Controlled Trials of BPA

Only two RCTs have been performed to date to evaluate the efficacy of BPA versus medical therapy alone. The French RACE trial [52] enrolled 105 patients who were randomly assigned to either BPA therapy alone or the pulmonary vasodilator Riociguat alone. At week 26, PVR reduction (*p* < 0.0001) was more pronounced in the BPA arm (66.7%) than in the Riociguat arm (39.9%). After week 26, patients whose CTEPH symptoms persisted had the opportunity to cross over (add on BPA after first-line Riociguat or add on Riociguat after first-line BPA), which allowed further PVR reduction in both groups. Notably, fewer adverse effects related to BPA were reported in patients who had been pretreated with Riociguat. A Japanese RCT, MR BPA [53], randomized 61 patients to compare BPA alone versus medical therapy alone with Riociguat using a primary endpoint of mPAP. After 12 months, a reduction of 16 mmHg was observed in the BPA cohort versus 7 mmHg in the Riociguat cohort (*p* < 0.0001). Both studies’ primary and secondary endpoints favored the BPA arm (PVR, mPAP, 6MWD, WHO functional class). Hemoptysis was the most frequently observed adverse effect in the BPA arm, occurring in 8% of the procedures in the RACE trial and 9% in the MR BPA trial. These RCTs demonstrate the efficacy of BPA alone in improving cardiopulmonary functional and hemodynamic parameters compared to medical therapy alone with Riociguat.

There were several major limitations of these open-labeled RCTs. They used a hemodynamic primary endpoint rather than a clinical primary endpoint. In the U.S., most patients undergoing BPA are already on PH-targeted medical therapy. These RCTs highly restricted two arms to a single therapy, which limits the generalizability of these trials, particularly in the U.S. [52,53]. The lack of definitive treatment goals for BPA possibly introduces bias by incentivizing researchers to continue BPA. Furthermore, the small sample size of these open-labeled studies makes these results prone to bias. Both studies excluded patients with persistent pulmonary hypertension following PTE, although both medical therapy and BPA have proven beneficial among this patient population [45,52]. The RACE trial criteria for add-on therapy (PVR > 4 WU) was not very ambitious as patients who adequately responded to their assigned therapy were ineligible to receive the crossover treatment, which could have further reduced their symptom burden. RACE trial authors suggest the next step is to test further the efficacy of add-on therapy among an expanded patient population with precapillary PVR of > 3 WU; therefore, further investigation is needed to explore the effects of multimodal, sequential combinations of medication and BPA.

## 9. Complications

Complications of BPA can be intraprocedural or postprocedural [17,54]. Potential complications during BPA include vascular injury (such as wire perforation, vascular dissection/perforation from balloon over-dilatation, or high-pressure contrast injection) with or without hemoptysis [17]. Vascular injury during BPA usually manifests as sudden cough, drop in oxygen saturation, and hemoptysis [29]. Another important complication following BPA is lung acute injury or reperfusion pulmonary edema (characterized by radiographic opacity, with or without hemoptysis and hypoxemia) [17]. The initially published U.S. BPA experience indicated a very high rate of major complications, such as mortality, hemoptysis, and the requirement for mechanical and positive pressure ventilation [20,21]. Subsequently, these complication rates have significantly improved with refinements in BPA techniques. Perioperative complications have decreased globally in recent years due to techniques such as using undersized balloons for initial dilations, incorporation of pressure wires, and increased operator experience [21]. The authors have reported their experience of the first 211 BPA sessions with an incidence RPE of 1.3% and a hemoptysis rate of 4.7% [21].

Hemoptysis is the most common complication of the BPA procedure and is mainly related to wire perforation or balloon injury [30]. Initial management of hemoptysis requires immediate balloon tamponade of the culprit vessel for 10–15 min [3,17,29,31]. Supplemental oxygen, oropharyngeal suctioning, cessation or reversal of anticoagulation with protamine, gel foam embolization, and repeat balloon tamponade are used as necessary [17]. If pulmonary hemorrhage persists despite these measures, mechanical ventilation with isolated lung ventilation, covered stent implantation, bailout transcatheter coil embolization, and extracorporeal membrane oxygen (ECMO) may be considered [17].

Reperfusion pulmonary edema typically presents hours after BPA revascularization, often accompanied by decreased oxygen saturation and pink frothy sputum [29]. Supportive care for RPE includes supplemental mask oxygen (moderate), non-invasive positive pressure ventilation with high-concentration oxygen inhalation (moderate to severe), and artificial ventilation (extremely severe) [54]. At our facility, a pressure wire is used to maintain pressure distal to the lesion below the 35 mmHg mean in patients with high pulmonary artery pressures (>35 mmHg) as this has been shown to reduce the incidence of RPE [21,54]. Utilizing undersized balloons and limiting the number of vessels treated per BPA session are also believed to reduce the incidence of RPE [3,17,31].

## 10. Knowledge Gaps

Despite recent advancements surrounding BPA techniques, several knowledge gaps must be addressed before BPA can be universally adopted. First, only a few surgical centers can perform PTE on distal lesions; therefore, an RCT will need to be conducted to further elucidate the comparative effectiveness of PTE surgery versus BPA in level III disease (fibrotic thromboembolic material starting at the segmental arterial branches) [3]. Furthermore, the RACE trial demonstrated the combined effect of Riociguat vasodilator therapy and BPA in reducing patient PVR. However, we still do not know if medical therapy is necessary for all BPA patients before the procedure. While anticoagulation therapy is standard with BPA, the ideal anticoagulation regimen before, during, and after the procedure has yet to be determined [28,37]. The necessity of BPA and PTE surgery among minimally symptomatic patients is unclear. Furthermore, inconsistencies remain in selecting primary efficacy endpoints. For instance, the RACE trial assessed PVR while the MR BPA trial assessed mPAP; both are hemodynamic rather than clinical endpoints. Moreover, BPA therapy is personalized to each individual patient, and multiple sessions are required to ameliorate their disease process [28]. Still, there must be a clear clinical endpoint to say we have completed the treatment course. The ESC/ERS guidelines suggest a minimum hemodynamic goal of mPAP < 30 mmHg; however, even this endpoint should be individualized, particularly in patients with multiple severe comorbidities [1,37]. While several studies have followed patients a few years after BPA, long-term outcomes are unavailable [37]. Most patients require multiple BPA sessions to achieve hemodynamic goals, but little is known regarding the ideal interval between BPA sessions. While rare, children at high risk for thromboembolism have been reported to develop CTEPH and have gone on to receive treatment with both PTE and medical therapy [55]. The role of BPA in this pediatric demographic remains largely unstudied. Regarding technical considerations, the role of pressure wire-guided vs. IVUS-guided BPA of lesions must be studied.

## 11. Future Perspectives

Technical and pharmacologic protocols, as well as outcome assessments surrounding BPA, must be standardized before we can truly determine whether adjunctive BPA can enhance clinical outcomes among inoperable CTEPH patients [2]. Going forward, dedicated angioplasty equipment that is specific to pulmonary circulation must be developed (i.e., tapered balloons, deflectable guides, and wires). We need to assess the potential role of stenting in proximal disease in which there may be severe recoil after balloon angioplasty alone [37].

We still lack prospective long-term data regarding the efficacy, safety, and durability of BPA procedures [3]. Moreover, most evidence supporting BPA stems from observational studies lacking sufficient controls and effective measures to reduce bias [2]. These studies have included a broad spectrum of CTEPH patients, spanning those with both operable and inoperable anatomy, due to significant variations in operability criteria across different centers [2].

Before we can confidently compare BPA to PTE for patients with segmental/subsegmental CTEPH, we need a multicenter assessment with objective determination of short-term success and evaluation of long-term outcomes of BPA [3]. Patients with symptomatic CTEPD without PH have not been adequately studied; in particular, the role of invasive and non-invasive cardiopulmonary exercise testing has not been adequately studied [37]. Moreover, pragmatic trial designs are crucial for the generalized application of this procedure, with specific emphasis on establishing qualifications for BPA practitioners and institutional standards for BPA programs [2]. Finally, the paucity of RCT data from the U.S. must be immediately addressed with multicenter U.S.-based RCTs.

## Figures and Tables

**Table 1:** Contemporary BPA data from the United States.

Outcomes at United States Centers	University of California [46]	Temple University [21]	University of Washington [47]	Mayo Clinic [48]	University of Michigan [49]
00Total no. of patients	97	77	30	31	18
Hemoptysis rate/sessions	9.2	4.7	17.8	4	3.8
Death (% of patients)	0	1.3	6.6	3.2	0
Change in mean PAP (mmHg)	−5.6	−6.4	−6	−11	−8.3
Change in PVR (Wood Units)	−1.2	−1.7	−1.9	−2.2	−1.7
Change in 6-min walk distance (m)	36.8	71.7	40	37	67.4

Abbreviations: PAP, pulmonary artery pressure; PVR, pulmonary vascular resistance. Note. Reprinted with permission from [2].

## Data Availability

Not applicable.
